# Noncontact respiratory monitoring during sleep: comparison of the touchless flow signal with respiratory inductance plethysmography flow (RIP_flow_) signal to assess respiratory events

**DOI:** 10.5664/jcsm.11486

**Published:** 2025-04-01

**Authors:** Paul S. Addison, Lara Brewer, Krishna M. Sundar, Robert Farney, Philip Smit, Andre Antunes, Dean Montgomery

**Affiliations:** ^1^ Research and Development, Patient Monitoring, Medtronic, Technopole Centre, Edinburgh, United Kingdom; ^2^ Anesthesiology Department, University of Utah, Salt Lake City, Utah; ^3^ Sleep Wake Center, University of Utah, Salt Lake City, Utah

**Keywords:** noncontact monitoring, respiratory signal, touchless monitoring, depth camera

## Abstract

**Study Objectives:**

A nonintrusive sleep monitoring technology based on the data from a commercially available depth sensing camera has been utilized for respiratory monitoring and shown to have good performance in assessing respiratory rates across a range of rates. This noncontact or *touchless*, technology allows continuous respiratory monitoring without attaching probes to the patient. We have noticed a strikingly similar morphology between the noncontact flow signal (NCM_flow_) and the flow signal derived using the chest and abdomen respiratory inductance plethysmography (RIP_flow_) bands. Here we report on a quantitative assessment of the morphological similarity between the two signals for a cohort of patients undergoing polysomnography.

**Methods:**

We collected depth data from 25 sleep patients undergoing attended polysomnography. Correlation and mutual trending were assessed between the NCM_flow_ and RIP_flow_ signals in individuals undergoing diagnostic and split night polysomnography using Pearson correlation and concordance between the NCM_flow_ and RIP_flow_ signals.

**Results:**

Strong correlation was observed across all patients between the NCM_flow_ and RIP_flow_ signals (range: 0.78–0.98, mean: 0.89 ± 0.06). Similarly, high values of concordance were achieved between the NCM_flow_ and RIP_flow_ signals (range: 0.85–1.0, mean: 0.96 ± 0.04).

**Conclusions:**

The high values of correlation and concordances confirm that the NCM_flow_ signal can potentially be used as a surrogate for RIP_flow_ signal during sleep. Our findings strongly support the potential for noncontact continuous monitoring of respiratory disturbances during sleep.

**Citation:**

Addison PS, Brewer L, Sundar KM, et al. Noncontact respiratory monitoring during sleep: comparison of the touchless flow signal with respiratory inductance plethysmography flow (RIP_flow_) signal to assess respiratory events. *J Clin Sleep Med*. 2025;21(4):713–721.

## INTRODUCTION

A common complaint about sleep studies is that the large number of wires and electrodes can make it difficult for patients to sleep.^1^ It is also labor intensive, costly and burdensome to place the full set of polysomnography (PSG) sensor probes.^2^^–^^4^ Although not an object of study in this work, pediatric population would also greatly benefit from cable reduction: safety issues associated with entanglement, especially in unattended studies, may also be of concern.^5^ In pediatric patients, sleep studies are limited due to removal of sensors and entanglement of wires,^6^ resulting in suboptimal PSG data.^7^ Additionally, children may also report anxiety when sleeping with wires.^8^ Respiration monitoring during a sleep study typically requires several measurement modalities, including an oronasal thermal airflow sensor to score apneas, a nasal pressure (NP) transducer to score hypopneas and sensors to monitor respiration effort, such as dual respiratory inductance plethysmography (RIP) chest and abdomen bands. There is a clear need to minimize the number of sensors and wires during attended PSG which has the potential to result in the improved ability to undergo a PSG with resultant benefits on sleep quality.

We have developed a noncontact monitoring (NCM) technology based upon data from a commercially available depth sensing camera. This *touchless* monitoring technology allows continuous respiratory monitoring without attaching probes to the patient. The depth sensing camera captures the distances (depths) from the camera to objects in its field of view and outputs a matrix of distances. Motion within the scene manifests as a change in depth across sequential frames. Depth cameras have been shown to be particularly adept at measuring small-scale coherent modulations in depths associated with respiratory activity of the patient.^9^ We have demonstrated the technology to be accurate in volunteers for continuously monitoring respiratory rate across a breathing range of 4–40 breaths per minute.^10^ This accuracy in respiratory rate monitoring was maintained even when participants were covered by a blanket.^11^ In addition to respiratory rate monitoring, we have identified distinct signal morphologies in the NCM respiratory waveform, including cyclical breathing patterns associated with an acute hypoxic challenge.^12^ Schätz et al.^13^ reported on the ability to detect sleep apnea events using a classifier trained on data from a range of commercially available depth cameras (including the Microsoft Kinect™, Redmond, WA, and Intel RealSense™, Santa Clara, CA, cameras). They obtained a sensitivity and specificity of 89.1% and 98.8%, respectively, when compared to classification of PSG breathing signal segments by a sleep specialist in a data set comprising 57 whole night PSG records. Yang et al.^14^ demonstrated the feasibility of detecting respiratory events in sleep patients by combining both depth sensing, using a Kinect™ camera (Microsoft), and audio inputs to a classifier. They found 0.4% error rates for identifying the classes (1) central apnea, (2) obstructive/mixed apnea, (3) hypopnea, and (4) other events, using a support vector machine.

For the NCM respiratory flow signal (NCM_flow_) to be a reliable respiratory monitoring signal in a clinical setting, the waveform should be morphologically similar to currently used signals such as RIP flow (RIP_flow_). The RIP_sum_ and RIP_flow_ signals are recommended for use by the American Academy of Sleep Medicine as alternatives to oronasal thermal airflow and NP transducer signals for identifying apneas and hypopneas when the primary airflow sensors are not functioning or the signals are not reliable.^15^ We hypothesized that the NCM_flow_ and RIP_flow_ signals would be quantitatively morphologically similar for sleep study patients. To measure this similarity we used two established methods when comparing the signals: Pearson correlation (R) and concordance (C).

## METHODS

### Patient cohort

This pilot study was conducted in accordance with the Declaration of Helsinki and all local regulatory requirements. The study was sponsored and funded by Medtronic. The protocol was approved by the Institutional Review Board at University of Utah Sleep-Wake Center (# 00146568). Written informed consent was obtained for all participants prior to study procedures.

Adult patients scheduled for a PSG to diagnose sleep-disordered breathing were eligible for the study. Patients were included for enrollment if they were ≥ 18 years of age. Patients were excluded if they received continuous positive airway pressure therapy or had contraindications to participate in this study due to any reason, including involvement in another study.

### Data acquisition

**Figure 1:**
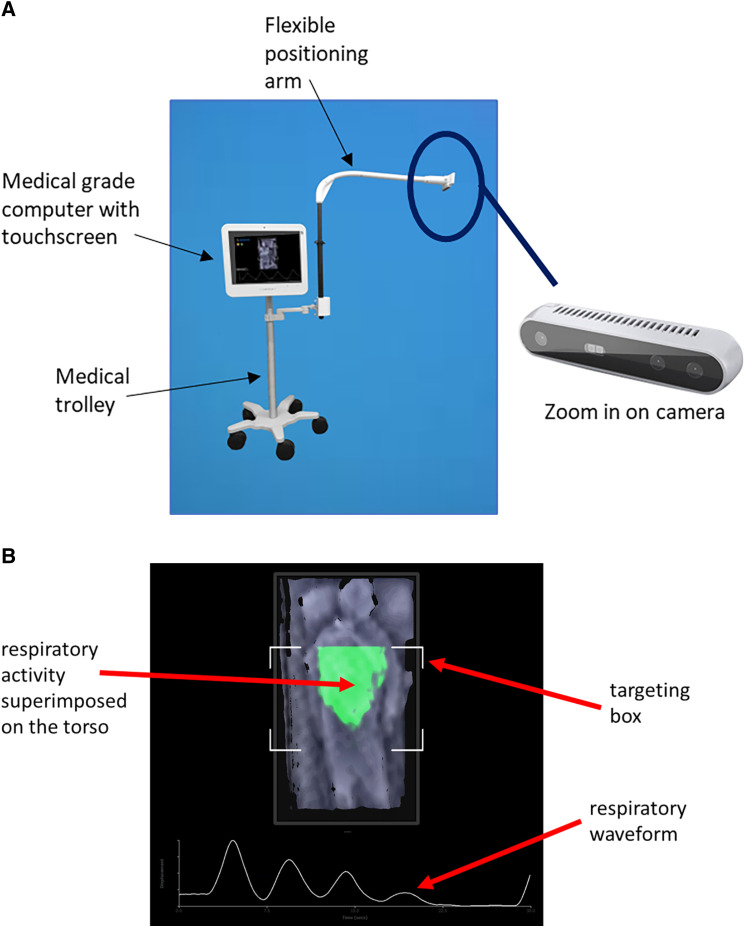
Collection device and respiratory visualization generated. **(A)** The prototype data collection device. **(B)** Respiratory visualization and waveform generated using the depth camera.

A prototype NCM system was used to acquire depth data at a rate of 15 Hz from the patient’s torso (**Figure 1A**) during the PSG. The NCM system comprises a camera (RealSense™ D415, Intel) suspended on a flexible arm attached to a trolley.^9^ Depth data were acquired by the camera placed approximately 1.1 m above the torso of the patient. The system required no calibration and the touchless respiratory signal output from the device (NCM_flow_) was used directly in the analysis described with no further postprocessing. Patients were allowed the option of blankets or coverings of their choice during the study, with most patients using some sort of covering for the majority of the night.

**Figure 1B** contains an example of a rendered depth image of an individual lying on a bed. The region of the detected respiratory activity is highlighted by a color patch superimposed onto the image. The frame-to-frame differences in depth associated with respiratory activity is integrated across the region of interest to derive a time-varying respiratory volume signal as shown below the depth image in **Figure 1B**. NCM_flow_ is calculated as the derivative of this signal. An example video clip from the NCM system is provided in the supplemental material.

Full overnight PSG (Natus, Sleepworks, Middleton, WI) recordings were obtained for each patient. The RIP chest and abdomen signals collected at 256 Hz were extracted from the PSG record and the associated RIP_sum_ and RIP_flow_ signals calculated according to American Academy of Sleep Medicine guidelines.^15^ The RIP_sum_ is the RIP_sum_ (belts), and excursions in the signal are an estimate of tidal volume. The RIP_flow_ is the time derivative of the RIP_sum_, and excursions in the signal are an estimate of airflow.^15^

### Data analysis methodology

Firstly, the RIP signals were synchronized with the NCM_flow_ signal where the offsets were adjusted and checked manually. However, we were not able to perform the synchronization over the whole sleep record for each individual due to nonlinear temporal shifting between the signals and intermittent data drops caused by hardware collection glitches. Although these data drops were of subsecond magnitude, they seriously impacted the synchronization and hence the R and C calculations. (For example, a quarter breath desynchronization could theoretically cause an R value to change from 1 to 0.) Given these constraints, the longest consistent period we could find for analysis across all patients was 30 minutes. For cases where more than 30 minutes were available, we selected the 30-minute segment with the most apnea/hypopnea events. This synchronization issue was due to a limitation of the acquisition software when comparing the PSG and the NCM signals, and affects mostly the correlation calculation, which is very sensitive to small shifts. It does not impact the clinical usefulness of the technology, as the correlation method is used only as a measure to compare similarity between signals from two different sources, and is not related to the depth technology. From a human user/clinician point of view, there is very little qualitative difference between the signals, even when the synchronization issue is present.

To quantitatively assess the morphological similarity between the NCM_flow_ and RIP_flow_ signals, we examined the correlation and concordance. The correlation is the normalized coefficient of covariance. The concordance is calculated by taking the difference in each signal (ΔNCM_flow_ and ΔRIP_flow_) occurring over a prespecified time scale and plotting these against each other. Therefore, if the signals trend exactly, all positive differences will match and all negative differences will match; thus all data will reside in the first and third quadrants of the concordance plot. Concordance is the proportion of data in the first and third quadrants. Concordance was measured over 2-second timescales that allowed for intra-breath morphology testing. To account for low level signal noise, 10% of the data were not included in the concordance calculation.

The global mean and standard deviation of the individual patient results were also calculated. The data were further stratified according to sleep positions observed over the whole cohort for the analyzed signals. Prior to the R and C calculations, the two signals were normalized by subtracting the mean value and dividing by the standard deviation. In addition, 1.54% of data was excluded due to motion-related effects on signals. No other data were excluded from the analysis. MATLAB™ software (R2023a, Natick, MA) was used for all data processing and statistical analysis.

## RESULTS

Of the 30 participants enrolled in the study, the data (NCM and/or PSG) was missing for 4 individuals (due to human error, the NCM signal acquisition was not started for 3 patients, and 1 PSG record was accidentally deleted during file transfer) and 1 individual withdrew from the study. Thus, data from 25 participants were available for analysis. Participant demographics are summarized in **Table 1**. Out of these 25 patients, 13 were female. Mean age was 44.9 ± 15.8 years (range: 20–81 years) with mean body mass index of 33.2 ± 10.0 (range: 21.1–62.3). The mean number of breaths analyzed per participant during the analysis period was 488 ± 125 (range: 223–755); the mean number of apneas was 4.6 ± 7.1 (range: 0–24) and for hypopneas was 13.2 ± 15.1 (range: 0–55). The average percentage of time spent in different sleep stages and positions were as follows: (1) sleep stages—N1 (9.6%), N2 (55.8%), N3 (6.8%), REM (18.2%), wake (9.6%); (2) sleep positions—supine (60.6%), prone (7.3%), right lateral (13.7%), left lateral (18.4%).

**Table 1:** Patient demographics together with number of breaths and respiratory events in each patient’s signal segment used in the analysis along with percentage of time in different body positions and sleep states.

ID	Sex	Age	BMI	Apneas	Hypopneas	Estimate Number of Breaths	Body Position (% of time)	Sleep Stage (% of time)
1	F	70	34.6	1	4	416	L(100)	N1(13), N2(79), N3(4), W(4)
2	F	24	23.2	2	0	532	S(100)	N2(78), N3(22)
3	F	52	37.1	3	30	502	S(100)	N2(26), N3(14), R(60)
4	F	20	21.2	1	1	436	L(100)	N1(7), N2(55), R(38)
5	M	50	31.4	18	35	223	S(100)	N2(97), W(3)
6	M	28	59.2	0	0	693	P(100)	N1(9), N2(89), W(2)
7	M	38	26.5	4	1	405	S(100)	N1(45), N2(38), W(17)
8	M	36	25.6	14	21	504	R(25), S(75)	N1(4), N2(89), W(7)
9	M	81	38.0	0	23	674	S(100)	N1(5), N2(74), W(21)
10	F	52	25.4	2	5	378	R(100)	N1(12), N2(83), W(5)
11	F	24	31.4	2	18	685	S(100)	N1(4), N2(8), R(88)
12	M	67	28.7	11	4	375	L(41), S(59)	N1(20), N2(42), W(38)
13	M	58	38.3	24	7	413	L(100)	N1(5), N2(43), N3(2), R(25), W(25)
14	F	53	28.0	1	0	419	S(100)	N2(98), R(2)
15	F	45	34.0	19	9	293	L(2), S(98)	N1(21), N2(33), W(46)
16	F	49	62.3	10	55	521	L(100)	N1(5), N2(95)
17	M	38	31.7	0	4	482	L(17), P(83)	N1(4), N2(41), R(43), W(12)
18	F	26	35.2	0	1	549	S(100)	N2(93), N3(7)
19	M	43	30.2	0	44	415	R(18), S(82)	N1(18), N2(55), W(27)
20	F	50	33.5	0	11	755	R(100)	N1(3), N2(55), N3(2), R(32), W(8)
21	M	36	35.7	2	28	538	S(100)	N2(1), R(89), W(10)
22	F	46	38.6	0	16	594	S(100)	N1(23), R(77)
23	F	30	21.1	0	0	479	S(100)	N1(5), N2(55), N3(30), W(10)
24	M	67	21.8	1	10	504	S(100)	N1(37), N2(59), W(4)
25	M	39	37.3	0	3	426	R(100)	N2(10), N3(90)

Body positions: L = left side, P = prone, R = right side, S = supine. Sleep stage: N1 = stage 1 non-rapid eye movement, N2 = stage 2 non-rapid eye movement, N3 = stage 3 non-rapid eye movement, R = rapid eye movement, W = wake. BMI = body mass index, F = female, ID = identification, M = male.

**Figure 2:**
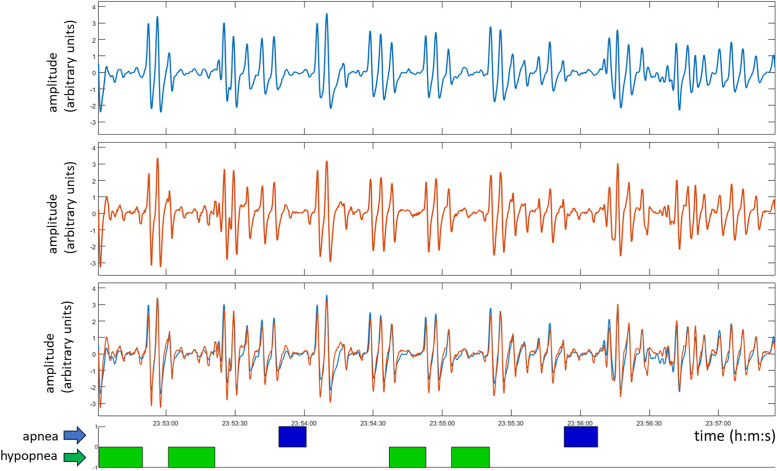
Example of the flow signals from the depth camera system and the RIP bands. NCM_flow_ (blue) and RIP_flow_ (red). The individual signals are shown above the overlaid plots of different flow signals. Apneas and hypopneas are shown below as blue and green patches, respectively. RIP = respiratory inductance plethysmography, NCM_flow_ = noncontact monitoring flow, RIP_flow_ = respiratory inductance plethysmography flow.

**Figure 2** contains a typical NCM_flow_ and RIP_flow_ signal captured during the trial. Examples of the scored events are shown below the signals. The number of breaths, apneas and hypopneas per signal segment are provided in **Table 1**, along with sleep state and position of individual patients.

**Figure 3:**
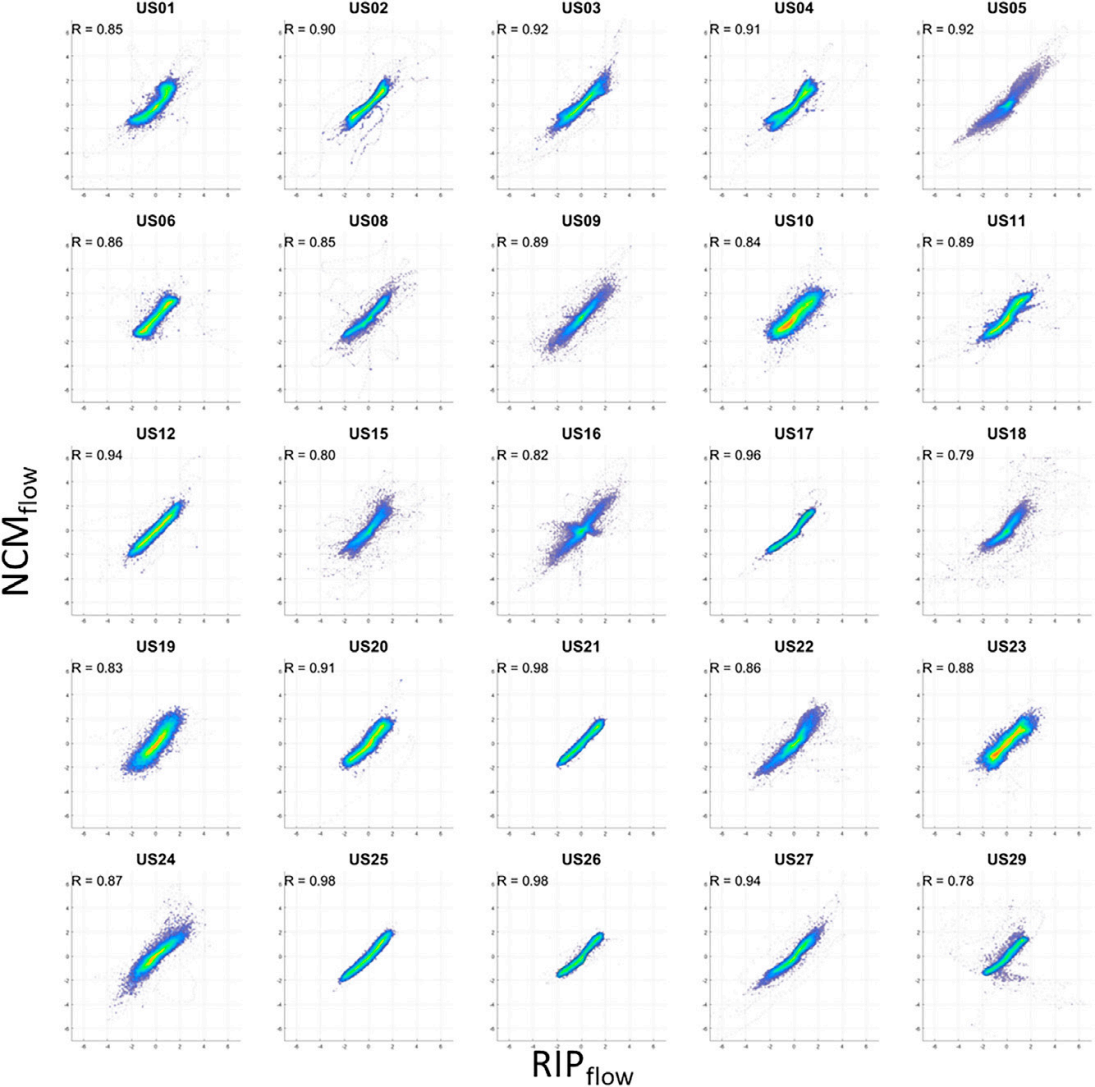
Scatterplots of noncontact monitoring derived flow NCM_flow_ against respiratory inductance plethysmography derived flow RIP_flow_ for all patients. NCM_flow_ = noncontact monitoring flow, R = Pearson correlation, RIP_flow_ = respiratory inductance plethysmography flow.

**Figure 4:**
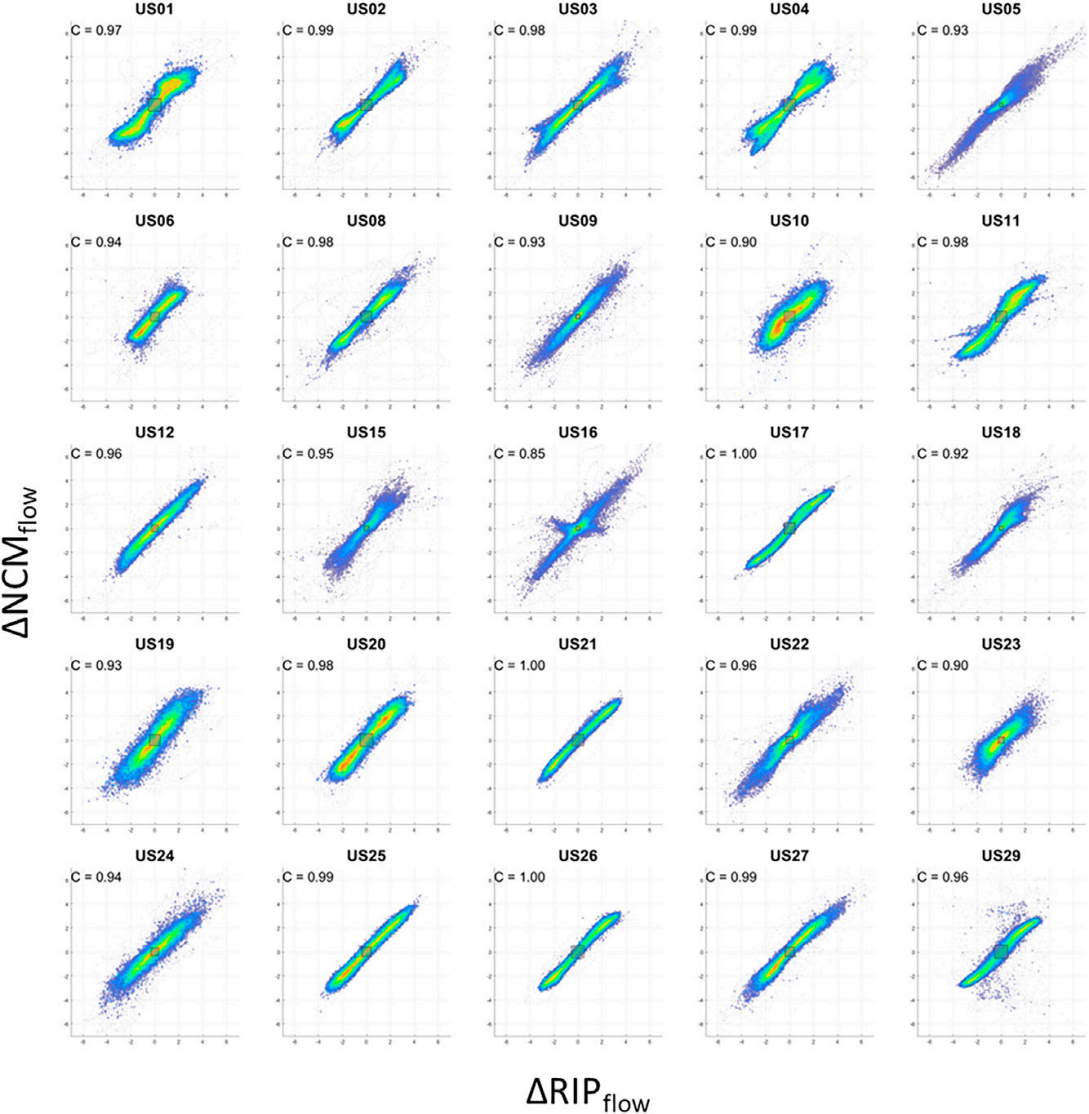
Concordance plots of NCM_flow_ against RIP_flow_ for all patients. C = concordance, NCM_flow_ = noncontact monitoring flow, RIP_flow_ = respiratory inductance plethysmography flow.

**Figure 3** contains individual participant scatter plots of NCM_flow_ against RIP_flow_, each displaying strong to very strong correlation between the two signals (range: 0.78–0.98, mean: 0.89 ± 0.06). A value of unity implies perfect correlation between each signal. **Figure 4** contains the corresponding concordance plots which again shows a strong concordance across all signals (range: 0.85–1.0, mean: 0.96 ± 0.04). A value of unity implies perfect cotrending behavior between the signals.

We performed several stratifications of the results to check for consistency, including categorizations based on age (< 50 years, ≥ 50 years), body mass index < 35, ≥ 35), apnea-hypopnea index (< 15 events/h, ≥ 15 events/h), and sex (**Table 2**). We found no significant differences based on these categorizations. The data were further stratified according to sleep positions and sleep states observed for the whole cohort.

**Table 2:** R and C values for the stratified analysis.

Variable		R	C
Age	< 50 years	0.89	0.97
	≥ 50 years	0.89	0.95
Body mass index	< 35 kg/m^2^	0.89	0.97
	≥ 35 kg/m^2^	0.88	0.94
Sex	Female	0.91	0.97
	Male	0.86	0.94
Apnea-hypopnea index	< 15 events/h	0.90	0.98
	≥ 15 events/h	0.87	0.92
Position	Side left	0.85	0.95
	Side right	0.85	0.95
	Supine	0.91	0.96
	Prone	0.88	0.96
Sleep stage	Stage 1 NREM	0.89	0.96
	Stage 2 NREM	0.90	0.96
	Stage 3 NREM	0.92	0.99
	REM	0.92	0.96
	Wake	0.76	0.94

C = concordance, NREM = non-rapid eye movement, R = Pearson correlation, REM = rapid eye movement.

## DISCUSSION

We have developed a noncontact *touchless*, sleep monitoring technology based on a depth-sensing camera. This technology allows continuous monitoring of respiratory disturbances without attaching probes to the patient. We tested the technology on a cohort of sleep study patients, where the similarities between the PSG RIP_flow_ signal and the touchless flow signal, NCM_flow_, were assessed quantitatively using correlation and concordance. This analysis between RIP_flow_ and NCM_flow_ was conducted for different body positions and sleep states. Strong correlations were observed across all patient signal pairs. The consistently high R values are indicative of the distinct morphological similarity between the signals. Concordance is used to demonstrate the cotrending behavior, rather than correlation, between signals. Hence, as a measure it does not require linear scaling. It is, however, a slightly less used statistic but has been applied in the trending analysis of a variety of physiological signals including tidal volume,^16^ blood pressure,^17^ oxygen saturation,^12^ stroke volume and cardiac output.^18^ High concordances between between RIP_flow_ and NCM_flow_ were obtained across individuals despite variations in sleep apnea severity, body mass index, age, or sex.

A limitation of the current analysis is the inability to perform correlation and concordance analysis for each individual over their entire sleep duration. As discussed in the Methods section, this limitation was due to nonlinear temporal shifting between the signals and intermittent data drops due to hardware collection glitches. Although this limited the maximum consistent length we could find for analysis across all patients to 30 minutes, it did comprise an average of 488 breaths per individual with an average of 18 apnea/hypopnea events per participant. In addition, the selected signal segments spanned all sleep states and sleeping positions (although note there is little intraindividual variation for position). Thus, the consistently strong R and C values obtained for all participants over this timescale is highly indicative of the consistency in the morphological similarity between the signals despite the signals originating from very different technologies. Another limitation of the analysis is that we have not shown its behavior during paradoxical motions due to obstructive breathing where out-of-phase chest-abdominal movements are present. The NCM_flow_ signal simply averages all motion across the torso region. However, it is worth noting that the RIP_flow_ signal also aggregates motions from the chest and abdomen and so the two signals are analogous to that extent. Future work should investigate the effect of paradoxical motions on the utility of the NCM flow signal, although it may be possible to segment out the chest and abdomen regions of the image. In general, more detailed analysis of the performance with respect to a range of respiratory signals should be conducted including Cheyne-Stokes respiration, central apneas, mixed apneas, hypopneas, etc.

We utilized the RIP_flow_ signal as comparison as it has been shown to represent airflow well during assessment of sleep-disordered breathing. Fortin et al. have shown statistical correlation between events scored using the RIP_flow_ signal and standard sensors used in PSG and suggested that RIP_flow_ could be a reliable method on its own for respiratory event scoring.^17^ They found statistical correlations between apnea-hypopnea index values determined by both standard PSG scoring and RIP_flow_ scoring when compared to a manual scored reference. Magalang et al. showed that RIP_flow_, NP, and a flow signal derived from NP (transformed NP) all produced extremely high intraclass correlation coefficients (all ≥ 0.96) for apnea-hypopnea index scoring when compared to manual scoring for home sleep apnea tests.^19^ These findings are further supported by the work of Eriksson et al. who found no statistical difference in respiratory event identification between calibrated RIP_flow_ and oronasal thermal flow and NP signals.^20^ Given these previous results, we used RIP_flow_ as a comparison to NCM_flow_ instead of the primary oronasal flow and NP signals.^15^ Based upon the morphological similarity we have demonstrated between RIP_flow_ and NCM_flow_, the touchless flow signal yields great promise as a valid respiratory monitoring modality during PSG.

## CONCLUSIONS

The high degree of the correlation and concordance reported confirm the strong match we observe visually between the NCM_flow_ and the RIP_flow_ signals. Our findings strongly support the potential for using a depth camera system to obtain noncontact continuous monitoring of respiratory disturbances during sleep. Utilizing noncontact respiratory monitoring allows for significantly less obtrusive sleep monitoring capability, which may be particularly beneficial in settings or populations where attaching sensors can be burdensome to the patient and can impede sleep. Future work will aim to determine the viability of developing a touchless sleep scoring methodology in clinical settings.

## DISCLOSURE STATEMENT

All authors have seen and approved the manuscript. This work was performed at the Sleep | Wake Center, University of Utah, Salt Lake City, Utah. Paul S. Addison, Philip Smit, Andre Antunes, Dean Montgomery are all paid employees of Medtronic who funded the study. The other authors were not paid to undertake the study and report no conflicts of interest.

## Supplemental Materials

10.5664/jcsm.11486 Supplemental Video 1

## ABBREVIATIONS

C: concordance
NCM: noncontact monitoring
NCM_flow_: noncontact monitoring flow
NP: nasal pressure
PSG: polysomnography
R: Pearson correlation
RIP: respiratory inductance plethysmography
RIP_flow_: respiratory inductance plethysmography flow

## EDITOR’S NOTE

The Emerging Technologies section focuses on new tools and techniques of potential utility in the diagnosis and management of any and all sleep disorders. The technologies may not yet be marketed, and indeed may only exist in prototype form. Some preliminary evidence of efficacy must be available, which can consist of small pilot studies or even data from animal studies, but definitive evidence of efficacy will not be required, and the submissions will be reviewed according to this standard. The intent is to alert readers of *Journal of Clinical Sleep Medicine* of promising technology that is in early stages of development. With this information, the reader may wish to (1) contact the author(s) in order to offer assistance in more definitive studies of the technology; (2) use the ideas underlying the technology to develop novel approaches of their own (with due respect for any patent issues); and (3) focus on subsequent publications involving the technology in order to determine when and if it is suitable for application to their own clinical practice. The *Journal of Clinical Sleep Medicine* and the American Academy of Sleep Medicine expressly do not endorse or represent that any of the technology described in the Emerging Technologies section has proven efficacy or effectiveness in the treatment of human disease, nor that any required regulatory approval has been obtained.

## OPEN ACCESS

Copyright 2025 The Authors. This is an open access article, distributed under the Creative Commons Attribution 4.0 International License. Sharing and adaptation are permitted provided attribution to its original publication in the Journal of Clinical Sleep Medicine is made in accordance with the license.
